# EVALUATION OF ESOPHAGEAL ACHALASIA: FROM SYMPTOMS TO THE CHICAGO CLASSIFICATION

**DOI:** 10.1590/0102-672020180001e1376

**Published:** 2018-07-02

**Authors:** Rafael Melillo LAURINO-NETO, Fernando HERBELLA, Francisco SCHLOTTMANN, Marco PATTI

**Affiliations:** 1 Departamento de Cirurgia, Escola Paulista de Medicina, Universidade Federal de São Paulo, SP; 2 Department of Surgery, University of North Carolina, Chapel Hill, North Carolina, USA

**Keywords:** Esophageal achalasia, Deglutition disorders, Endoscopy, Digestive system, Manometry., Transtornos de deglutição, Técnicas de diagnóstico do sistema digestório, Endoscopia do sistema digestório, Manometria.

## Abstract

***Introduction*:**

The diagnosis of achalasia may be suggested by clinical features but a complete work-up is required not only to confirm the diagnosis but also to grade the disease by severity or clinical subtype.

***Objective*:**

To review the current evaluation of esophageal achalasia and its correct comprehension.

***Method*:**

The literature review was based on papers published on Medline/Pubmed, SciELO and Lilacs, crossing the following headings: “esophageal achalasia”; “deglutition disorders”; “diagnostic techniques”, “digestive system”; “endoscopy, digestive system”; “manometry”.

***Results*:**

The diagnosis of achalasia is suggested by clinical features but is not sufficient to distinguish this from other esophageal disease. It must be confirmed by further diagnostic tests, such as esophagogastroduodenoscopy, barium swallow and manometry. Recent advances in diagnostic methods, including high resolution manometry might even help predicting outcome or selected more appropriate procedures to treat the disease.

***Conclusion*:**

A detailed and systematic study of achalasia patients allows not only a correct diagnosis but also contributes to therapeutic decision making and prognosis.

## RESUMO

***Introdução*:**

O diagnóstico da acalásia pode ser sugerido pelo quadro clínico; porém, completa investigação se faz necessária não apenas para confirmar o diagnóstico, mas, também, para estratificar a doença quanto à gravidade ou sub-tipo clínico.

***Objetivo*:**

Revisar os atuais métodos diagnósticos da acalásia do esôfago e sua correta interpretação.

Revisão da literatura realizada nas bases de dados Medline/Pubmed, SciELO e Lilacs, cruzando-se os descritores “acalásia esofágica”, “transtornos de deglutição”, “técnicas de diagnóstico do sistema digestório”, “endoscopia do sistema digestório” e “manometria”.

***Resultados*:**

O diagnóstico da acalásia é sugerido pelo quadro clínico, o qual, no entanto, é insuficiente para diferenciar esta doença de outras afecções esofágicas. O diagnóstico deve ser confirmado por endoscopia digestiva, estudo radiológico contrastado e manometria. Recentes avanços nos métodos diagnósticos, incluindo a manometria de alta resolução, podem também auxiliar no estabelecimento do prognóstico da doença ou na escolha da melhor modalidade de tratamento a ser realizada.

***Conclusão*:**

Estudo detalhado e sistemático dos pacientes portadores de acalásia permite não apenas diagnóstico correto, mas também contribui na escolha da melhor opção terapêutica e estabelecimento do prognóstico destes indivíduos.

## INTRODUCTION

Achalasia is a rare primary esophageal motility disorder that occurs with equal distribution irrespective of gender and race, but with increasing incidence with age and variable prevalence in different countries^1^. Although etiology is still elusive, achalasia pathophysiology, diagnosis and treatment is relatively well understood^18^. Achalasia is predominantly an idiopathic disease secondary to a selective loss of inhibitory neurons of the myenteric plexus, most likely due to an autoimmune phenomenon in response to unknown antigens^14^. Similar clinical presentation, however, can occur in patients with pseudoachalasia (5% of patients with suspected achalasia) due to malignant obstruction^4^ or operations^20^ at the esophagogastric junction. Achalasia can also be secondary to a tropical disease called Chagas´ disease, characterized by degeneration of the myenteric plexus due to *Trypanosoma* infection^6^.

The diagnosis of achalasia is suggested by clinical features and confirmed by further diagnostic tests, such as esophagogastroduodenoscopy, barium swallow and manometry^4^. These exams are not only used to establish the diagnosis but are also helpful to grade the disease by severity or clinical subtype. Recent advances in diagnostic methods, including high resolution manometry, might even help predicting outcome^23,25^ or selected more appropriate procedures^10^. Achalasia diagnosis is sometimes delayed and confused with gastroesophageal reflux disease due to low suspicion of the illnesses and underuse of esophageal manometry^15^.

This study aimed to review the current evaluation of esophageal achalasia and its correct comprehension.

## METHOD

The literature review was based on papers published on Medline/Pubmed, SciELO and Lilacs, crossing the following headings: Esophageal achalasia; Deglutition disorders; Endoscopy; Digestive system; Manometry.

## RESULTS

### Clinical presentation

Dysphagia and regurgitation are the most common symptoms. Dysphagia may initially be noticed for solids only, but as many as 70-97% of patients with achalasia have dysphagia for both liquids and solids at presentation. The regurgitation of undigested, retained food occurs in about 75% of these patients^27^.

Other symptoms include chest pain that is experienced by nearly 40% of patients with it, which must be differentiated from angina pectoris of cardiological origin. About 60% of achalasia patients may have some degree of weight loss at presentation due to poor esophageal emptying and decreased or modified food intake^27^.

The most common extraesophageal manifestations are pulmonary complications. Structural or functional pulmonary abnormalities occur in more than half of patients, and might be due to recurrent aspiration or tracheal compression from a dilated esophagus^4^. Chagas´ disease may affect other target organs such as the colon and the heart^6^. 

There are different scores to quantify the severity and frequency of symptoms. The Eckardt symptom score is the grading system most frequently used for the evaluation of symptoms, stages and efficacy of achalasia treatment. It attributes points (0 to 3 points) for four symptoms of the disease (dysphagia, regurgitation, chest pain and weight loss), ranging from 0 to 12. Scores of 0-1 corresponds to clinical stage 0, 2-3 to stage I, 4-6 to stage II, and a score >6 to stage III ^5^ (Table 1).

**TABLE 1:** Eckardt score for symptomatic evaluation in achalasia^5^

Score	Weight loss (kg)	Dysphagia	Retrosternal Pain	Regurgitation
0	None	None	None	None
1	< 5	Occasional	Occasional	Occasional
2	5-10	Daily	Daily	Daily
3	> 10	Each meal	Each meal	Each meal

Symptoms only, however, do not reliably diagnose the disease since there is an overlap of symptoms with other esophageal diseases, particularly gastroesophageal reflux disease. Furthermore, symptoms presence or severity does not correlate with manometric findings, degree of esophageal dilatation or prognosis. A complete workup is necessary in these patients, not only for the diagnosis but for prognosis and to establish the proper therapeutic approach.

### Upper digestive endoscopy

Endoscopy may suggest the diagnosis of achalasia, but has low accuracy. The esophageal body may appear dilated, atonic, and often tortuous at endoscopy in more advanced degrees of achalasia. Some resistance to trespass the cardia may be noticed. Esophageal mucosa may be normal but esophagitis with friability, thickening, and even erosions may be noticed secondary mainly to chronic stasis^27^.

Upper endoscopy must be performed in all patients with dysphagia and suspected achalasia. The main reason is to rule out esophageal cancer, or the development of pre-malignant or malignant lesions secondary to chronic stasis. Pseudoachalasia results from tumors at the esophagogastric junction and mimic classic achalasia, although clinical differences, such as older patients, greater weight loss and shorter duration of symptoms are seen. These tumors may be missed endoscopically in up to 60% of patients with pseudoachalasia due to a submucosal presentation. Endoscopic ultrasonography and CT scan may prove useful in patients with non-diagnostic endoscopy, and high degree of clinical suspicion for pseudoachalasia, but it are not recommended as a routine tests in achalasia^26^ ^,^ ^28^. Achalasia is an important risk factor for esophageal cancer with an incidence of up to 9% of cancer developing in achalasia series^8^ or 10-50 times higher than the general population^2^. 

### Barium swallow

**FIGURE 1:**
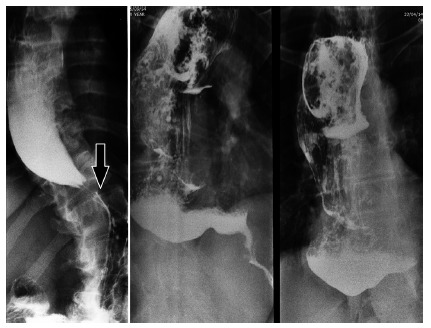
Barium swallow in achalasia (proximal dilated esophagus, distal taper- arrow)

It is important to define the morphology of the esophagus (diameter and axis) and associated conditions, such as epiphrenic diverticula or cancer. Classical findings are the distal esophagus tapering in a “bird’s beak” configuration with proximal dilation of the organ, sometimes with an air-fluid level, and absence of intra-gastric air. In more advanced cases, severe dilatation with stasis of food and a sigmoid-like appearance can occur^14^ (Figure 1). However, dilation of the esophagus may be absent, and the organ may appear normal, especially during the early stages of the disease^1^ ^,^ ^14^. A classification for the degree of esophageal dilatation is in use by Latin American surgeons due to the frequent finding of dilatation in Chagas´ disease^21^(Table2).

**TABLE 2:** Classification for esophageal dilatation based on barium esophagogram according to Rezende^21^

Maximum esophageal diameter (cm)	Grade
<4	I
4-7	II
7-10	III
>10	IV

Timed barium swallow can be performed to assess emptying of the esophagus, by measuring the height of the barium column 5 min after ingestion of diluted barium^3^.

### Manometry

Esophageal manometry defines the diagnosis of the disease with a very high level of certainty, even in the very early stages of the disease. The manometric picture of achalasia is characterized by failure of the lower esophageal sphincter (LES) to relax during swallowing and aperistalsis^18^.

**FIGURE 2:**
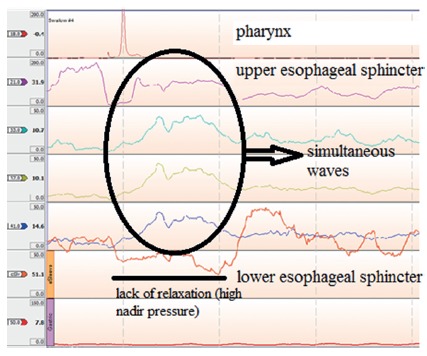
Conventional manometry in a case of achalasia

Conventional manometry has some technical limitations that allow the measurement of LES relaxation based on the nadir pressure during swallow. In this setting, about 70-80% have absent or incomplete LES relaxation with wet swallows, while the remainder will have a nadir pressure within normal limits but with short duration relaxation (<6 s). Aperistalsis is usually noticed as simultaneous mirrored contractions with complete loss of propagation of the contractions (Figure 2). In advanced cases, pressurization of the esophagus from incomplete evacuation of air and retained food may be seen. Hypertonic LES was considered one of the criteria for the diagnosis although this is found in only half of patients with achalasia. A subset of patients presented with high amplitude simultaneous waves, defined as vigorous achalasia^27^ ^,^ ^22^.

**FIGURE 3:**
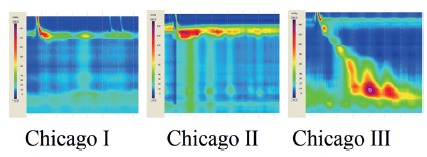
Achalasia subtypes in high resolution manometry

The introduction of high resolution manometry has improved the ability to diagnose achalasia and identify newer variants. More detailed parameters were created based on technological improvements^12^. LES relaxation is measured more precisely by the Integrated Relaxation Pressure that corresponds to the mean pressure of 4 s of greatest post-deglutitive relaxation in a 10 s gap, triggered at the beginning of a swallow^13^. Esophageal body analysis allowed to categorize achalasia into three groups (or variants), a classification known as the Chicago Criteria^23^, now in its 3.0 version^9^ (Table 3). These groups are characterized by pressurization of the esophageal body or not, and the presence of spastic contractions (Figure 3). 

**TABLE 3:** Manometric Chicago Classification for achalasia

Type	Lower esophageal sphincter	Esophageal body
I	Incomplete relaxation	Aperistalsis and absence of esophageal pressurization
II	Incomplete relaxation	Aperistalsis and panesophageal pressurization in at least 20% of swallows
III	Incomplete relaxation	Premature (spastic) contractions with distal contractility integral (DCI) >450 mmHg·s·cm with ≥20% of swallows

The Chicago Classification made major contribution for the prognosis of the disease. The subgrouping of achalasia types has direct relationship with outcomes with positive treatment response in 96% of cases in type II, 56% of type I and only 29% of those classified as type III^17^. A recent meta-analysis that encompassed nine studies and almost 730 patients^16^, found a difference in prognosis with different types of treatments such as pneumatic dilatation and surgical myotomy. The same pattern occurs after botulinum toxin injection^17^. 

The selection of the best initial approach for achalasia also appears to be influenced by the Chicago Classification subgrouping. While in types I and II, more conservative treatments such as pneumatic dilatation and surgical myotomy appear to be good options, type III seems to be better managed with per oral endoscopic myotomy than surgical myotomy, probably due to the ability to perform longer myotomies^7^ ^,^ ^11^.

## CONCLUSION

Patients with suspected achalasia must be evaluated with a complete work-up. Symptoms are not sufficient to distinguish achalasia from other esophageal disease. Furthermore, a detailed and systematic study of these patients allows not only a fast and correct diagnosis but also contributes to therapeutic decision making and prognosis. 
